# Supplementary material for: Wiedemann–Steiner Syndrome: Case Report and Review of Literature
Source: Children (Basel). 2022 Oct 12;9(10):1545. doi: 10.3390/children9101545 (PMC9600770; doi:10.3390/children9101545)
Supplement: Supplementary file 1 [file children-09-01545-s001.zip › children-1912335-supplementary.pdf]

Table S1: Detailed clinical and molecular information of WSS patients from the literature

Abbreviations: F: female; M: male; m: month; y: year; + : present; - : absent; ?: questionably present

Wiedemann-Steiner syndrome patients with detailed clinical data and mutation in KMT2A

**Study**	**Evans and Novais 2022**		**Wang et al. 2021**	**Nardello et al. 2021**	**Luo et al. 2021**			**Lee et al. 2021**	**Lee et al. 2020**		**Hirst and Evans 2021**	**Demir et al. 2021**
Sample ID	1	2			Patient 1	Patient 2	Patient 3		MCD-073	MCD-057		
Gender	F	F	F	M	F	F	F	M	M	F	F	F
Age (or age at last examination)	15y	12y	1y3m	9y	10y	5m	1y8m	5y	7y2m	4y11m	7y	8m
Country/Race	Caucasian	Caucasian	Chinese	Caucasian	Chinese	Chinese	Chinese	Chinese (Taiwan)	Korean	Korean		
Inheritance	De novo		De novo	De novo	De novo	De novo	De novo	De novo		De novo		De novo
cDNA			c.3566G>T	c.4433delG	c.883A>T	c.4171C>T	c.3499T>C	c.4696G>A	c.10217C>G	c.2552_2553del	c.3082A>T	c.3647_3650delAAGA
Protein			p. Cys1189Phe	p. Arg1478LeufsTer108	Lys295*	Gln1391*	Cys1167Arg	p. Gly1566Arg	p. Ser3406Ter	p. Lys851ArgfsTer14	p. Lys1028Ter	p.Lys1216ArgfsTer18
Current height			73.3cm (-2SD to -1SD)			59cm(<-2SD)	69cm(<-3SD)	104.9cm (19th, Z score =−0.55)				65cm(10-25th)
Current weight			7.5kg (-3SD to -2SD)			5kg(<-3SD)	10kg(<-2SD)	16.6kg (20th, Z score =−0.75)				5.68kg(<3rd)
Current head circumference			42cm (<-3SD)	53cm (z 1.4)								40cm(<3rd)
**Growth**												
Prenatal growth retardation			+	+	-	+	+	-			-	+
Postnatal growth retardation	+	+	+	+	+	+	+	+			+	+
Developmental delay	+	+	+	+	+	+	+	+	+	+	+	+
**Craniofacial features**	Mild facial dysmorphism	Notable facial features										
Microcephaly			+		-	-	+		+	+		+
High forehead			+		-	+	+					
Low hair line		+	-, high hair line		-	-						
Narrow palpebral fissures			-	+	-	+					+	
Downslanting palpebral fissures			-	+	-	+						
Hypertelorism			+		+	-		+	+			+
Strabismus			+									-
Eversion of lateral third of lower eyelids			+									
Long eyelashes			+	+	+	+						+
Ptosis			-			+						+
Broad and arching eyebrows			+	+								
Synophrys			+									+
Depressed nasal bridge					-	+		+				+
Wide nasal bridge			+		+	+						+
Broad nasal tip			+									
Bulbous nose			+									

Long philtrum			+		-	+					+	+
High palate			-									+
Cupid's bow, exaggerated			+									
Low-set ears				+	-	+				+		
Downturned corners of the mouth			+	+	+	+						+
Thin upper lip					-	+						+
Dental/oral anomalies											+	
Other Craniofacial features		Webbed neck	Full cheeks	Coarse hands, scaphocephaly	Mild facial asymmetry, epicanthus tarsalis			A mildly coarse face with mild mid-face hypoplasia, mild trigonocephaly, an always-open mouth with a mild tongue protrusion	Long nasal bridge, short philtrum	Hypotonic face, white face	Telecanthus,flared medial eyebrows, long palpebral fissures, and bilateral ear pits	Short nose, micrognathia
**Skeletal anomalies**												
Advance bone age	-	-			-							
Delayed bone age	+	-			+							
Scoliosis	mild spinal asymmetry		-									
Small and puffy hands and feet			+		-	+						
Brachydactyly			+									+
Clinodactyly			-	+								+
Rib anomalies			NA									
Sacral dimple		-	-	+							+	
Other skeletal anomaly	Notable in-toeing and internal foot progression angle, external tibial torsion and valgus flatfoot deformity, hip subluxation	Cervical fusion, basilar invagination, and associated Chiari type I malformation	Fetal pads, fat pads anterior to calcanei				Bilateral foot eversion		Skeletal anomaly		Bilateral hip dysplasia	Tapered fingers, a single transverse palmar crease in her left hand
**Hairiness**					-	-	-					
Hypertrichosis cubiti			+	+				+				-
Hypertrichosis of the back			+					+				+, Lumbar and coccygeal regions
Hypertrichosis of limbs	+		+			+		+				
Hypertrichosis, generalized		+	+		+	+	+	+	+		+	+
Thick hair			+		-	-		+				
Thick eyebrow												+
**Development and neurology**												
Hypotonia			+		-	+	+		+	+		+
Intellectual disability			+	+	+	+	+		+			
Speech delay/absence	+			+			+					
Seizures			-		-	-	-		-	-		

Behavioral disorder		Autism, attention deficit disorder		+					+	+	+	
**Organic problems**												
Cerebral				Cortical anomalies	Increased pituitarium	Widened bilateral fissure and anterior longitudinal division	Enlarged subarachnoid, choroid plexus cysts		Hypoplastic pituitary gland			
Ophthalmologic examination		Surgery for strabismus			Astigmatism						Intermittent exotropia and a right convergent squint which self-corrected	
Cardiac		A history of bicuspid aortic valve	-	A dilated (left ventricle) cardiomyopathy		Congenital cardiac malformation, endocardial fibroelastosis, patent ductus arteriosus and patent foramen ovale			-			
Renal			-	Cross ectopia of the left kidney, pyelectasis, bilateral megaureter and left hydrocele					-			
Feeding difficulties			+		-	+	+		-		+	
GH deficit		-										
Other		Premature adrenarche, middle ear cyst removal			Poor learning ability in mathematics, severe sensory integration dysfunction, as well as defective communication and socialization skills, IGF-1 deficiency	Recurrent infections, cytomegalovirus infection			Severe constipation	Cyclic vomiting		

Wiedemann-Steiner syndrome patients with detailed clinical data and mutation in KMT2A

**Study**	**Di Fede et al. 2021**						**Bruno et al. 2021**	**Mendoza 2020**	**Jezela-Stanek et al. 2020**	**Grangeia, Leao et al. 2020**	
Sample ID	RSTS-208	RSTS-250	RSTS-187	RSTS-251	RSTS-243	RSTS-221	10		Patient 3	Patient 1	Patient 2
Gender	M	M	M	F	M	M	M	M	F	F	M
Age (or age at last examination)	13y	16y	17y	25y	26y	9y	21y	3y	4y	11y	17y
Country/Race								Caucasian		Portugal	Portugal
Inheritance	De novo	Unknown (unavailable parents)	De novo	De novo	De novo	Unknown(unavaila ble parents)	De novo	De novo	De novo	De novo (father died)	De novo
cDNA	c.553C>T	c.1697_1717dup	c.3596G>A	c.3603del	c.3632_363	c.3897_3900dup	c.478C > T		c.4012+2T>A	c.8092del	c.839_843del
Protein	p.Arg185*	p.Leu566_Leu57 2dup	p.Trp1199*	p.Ser1202Profs*12	+2del p.?	p.Leu1303Serfs24*	p.(Arg160*)		p.IVS7 ds T-A +2	p.Glu2698Asnfs*59	p.Pro280Glnfs*3
Current height					164.8 cm (3rd centile)					142cm(25-50th)	152cm(<3rd)
Current weight										58kg(>97th)	45kg(<3rd)
Current head circumference										54cm(50th)	53cm(25-50th)
**Growth**											
Prenatal growth retardation	-	-	-		-			+	-	+	+
Postnatal growth retardation	+	-	+/-		+	+		+	+	-	+
Developmental delay			+		+	+		+	+	+	+
**Craniofacial features**											
Microcephaly	-	+	-		-	+			+	-	
High forehead											
Low hair line			+								
Narrow palpebral fissures	+	+	-	+	+	+			+		
Downslanting palpebral fissures	+	+	-	+	+	+					+
Hypertelorism	+	-	-	+	-	+			+	+	
Strabismus	-	-	-	-	+	-					+
Eversion of lateral third of lower eyelids											
Long eyelashes	+	+	+	+	+	+					
Ptosis	+	+	-	+	+	-				+(left> right)	+
Broad and arching eyebrows						+					
Synophrys	-	-	+	-	+	+					+
Depressed nasal bridge	+	+	-	-	+	-			-		
Wide nasal bridge	+										
Broad nasal tip									+		+
Bulbous nose			+								+
Long philtrum						-					
High palate	+			-	+	+			+	+	+
Cupid's bow, exaggerated											

Low-set ears	+	+	+	-	-	-				+	
Downturned corners of the mouth											
Thin upper lip	+	+		+	+	+					
Dental/oral anomalies	-	-			+	-			+		
Other Craniofacial features	Columella below the alae nasi, grimacing smile, prominent eyes.	Micrognathia, columella below the alae nasi	Columella below the alae nasi, grimacing smile, upturned corners of mouth, telecanthus	Columella below the alae nasi, epicanthal folds, high nasal bridge, upturned corners of mouth	Micrognathia, broad distalfingertips, telecanthus	Micrognathia, columella below the alae nasi, grimacing smile, abnormal ears, coarse face, low anterior airline, narrow forehead, convex nasal bridge, short philtrum	Morsus inversus, ogival palate, bilateral epicanthus		Epicanthus, wide nose, anteverted nares, retrognathia, open mouth	Trigonocephaly, telecanthus and bilateral epicanthus, micrognatia	Morsus inversus, ogival palate, bilateral epicanthus
**Skeletal anomalies**											
Advance bone age										-	-
Delayed bone age	-		-		-	-				-	-
Scoliosis				+		+					
Small and puffy hands and feet											
Brachydactyly	-	-	-	-	-	+			+		
Clinodactyly	-	-	-	-	+	-			+		
Rib anomalies											
Sacral dimple			+								
Other skeletal anomaly	Broad halluces, angulated thumbs, C2–C3 vertebral fusion	Broad halluces, broad thumbs	Broad thumbs and halluces		Broad halluces, broad thumbs	Broad halluces and kyphosis, broad and angulated thumbs	Tapered fingers		Tapered fingers, finger joint hyperextensibility, joint hypermobility, increased nuchal translucency, breech presentation, 2-3 finger syndactyly (slight), single tranverse palmar crease, deep palmar creases, abnormality of the fingernails (small), abnormality of the distal phalanx of finger, sandal gap, abnormality of the hallux	Turricephaly, round face	Clubfoot deformity, pectus excavatum
**Hairiness**											
Hypertrichosis cubiti											
Hypertrichosis of the back									+		+
Hypertrichosis of limbs										+	+
Hypertrichosis, generalized	-	-	+		+	+				+	+
Thick hair									+(Localized hirsutism)	+	+
Thick eyebrow	+	-	+	+	+	-	+				+
**Development and neurology**											
Hypotonia	-		-		+	+		+	+		
Intellectual disability	+	+	+	+	+	+			+		+
Speech delay/absence		+	+	+	+	+	+	+	+		
Seizures	-		-	-	-	-					

Behavioral disorder	+	+	+	?	+			+			
**Organic problems**											
Cerebral	Cerebellar vermis hypoplasia	Epileptiform abnormalities in the left fronto-temporal region		Craniocervical junction anomalies with a mild basilar impression.		-	Thin corpus callosum		Bilateral frontal polymicrogyria	Partial agenesis of the posterior corpus callosum	Thin corpus callosum
Ophthalmologic examination										Hypermetropia and astigmatism	
Cardiac	-	-	-		-	-	Ventricular septal defect	Ventricular septal defects (treated)	+		
Renal						-				Horseshoe kidney, normal renal function	
Feeding difficulties	-		-		+	+		+			
GH deficit						+					
Other	Organomegaly	Early puberty (treated)	Gastroesophageal reflux		Hearing loss, early puberty (treated)		Respiratory distress	Physical therapy management	Hyperbilirubinemia in the Neonatal period, gastroesophageal reflux, laryngeal stridor	Recurrent respiratory tract infections, adenoidectomy for snoring and obstructive sleep apnea, constipation.	Submucous palate cleft

Wiedemann-Steiner syndrome patients with detailed clinical data and mutation in KMT2A

**Study**	Giangiobbe et al. 2020									
Sample ID	Pt.1	Pt.2	Pt.3	Pt.4	Pt.5	Pt.7	Pt.8	Pt.9	Pt.10	Pt.11
Gender	F	F	M	M	M	F	F	M	F	M
Age (or age at last examination)	24y	4y(20m)	6y(4y)	14y(12y)	6y(5y)	5y(3y11m)	10y(6.5y)	19y(18y)	20y(18y)	17y
Country/Race										
Inheritance										
cDNA	c.2513G>A	c.4436G>C	c.3473G>A	c.2318dupC	c.7187_7191dupCAGAT	c.1588dupA	c.3460C>T	c.9857_9858delCC	c.5812C>T	c.6637delA
Protein	p.W838*	p.C1479S	p.C1158Y	p.S774Vfs*12	p.E2398Qfs*10	p.R530Kfs*12	p.R530Kfs*12	p.P3286Qfs*7	p.R1938*	p.M2213Cfs*4
Current height										
Current weight										
Current head circumference										
**Growth**										
Prenatal growth retardation	-	+	+	+	-	-	+	+	-	-
Postnatal growth retardation	+	+	+	+	-	+	+	-	+	+
Developmental delay	+	+	+	+	+	+	+	+	+(mild)	+
**Craniofacial features**										
Microcephaly	-	+	+	+	-	-	+	+	-	-
High forehead										
Low hair line										
Narrow palpebral fissures	+	+	+	-	+	+	-	+	-	+
Downslanting palpebral fissures	-	-	-	+	+	+	+	+	+	-
Hypertelorism	+	+	-	+	+	-	+	+	+	+
Strabismus	+	-	+	+	-	-	-	-	-	+
Eversion of lateral third of lower eyelids										
Long eyelashes	-	-	+	+	+	+	+	+	+	+
Ptosis										
Broad and arching eyebrows										
Synophrys										
Depressed nasal bridge										
Wide nasal bridge	+	+	+	+	+	-	+	+	+	+
Broad nasal tip	+	+		+		-	+	+	+	+
Bulbous nose										
Long philtrum										
High palate	-	-	-	+	+			+	+	+
Cupid's bow, exaggerated										
Low-set ears	-	-	+	+	+	-	+	-	-	+

Downturned corners of the mouth										
Thin upper lip	-	+	+	-		+	+	+	-	+
Dental/oral anomalies	-	-	+	-	+		-		+ (supernumerary teeth)	-
Other Craniofacial features	Round and flat face	Round and flat face	Round and flat face			Round and flat face	Round and flat face	Round and flat face	Round and flat face	
**Skeletal anomalies**										
Advance bone age										
Delayed bone age				-		-		-	-	
Scoliosis				+				+, mild	+, L1-L2 kyphosis (mild),schmorl's nodes of the T11, T12, L1, L2 and L3 endplates	
Small and puffy hands and feet										
Brachydactyly	-	-	+	+	+	+, thumbs only	+	+	+	+
Clinodactyly	-	+	+	+	+	+	+	+	+	+
Rib anomalies		+,10 pairs of ribs			+, 11 thoracic vertebrae, short coccyx				+, bilateral ribs hypoplasia in T1	+, cervical ribs in C7
Sacral dimple	-	+	+	+	+	+	-	+	-	+
Other skeletal anomaly	Craniovertebral junction anomalies	Craniovertebral junction anomalies	Craniovertebral junction anomalies	Short, thick limbs, craniovertebral junction anomalies	Short, thick limbs, craniovertebral junction anomalies	Craniovertebral junction anomalies	Craniovertebral junction anomalies	Short, thick limbs, craniovertebral junction anomalies	Craniovertebral junction anomalies	Short, thick limbs, coccyx cyst, craniovertebral junction anomalies
**Hairiness**										
Hypertrichosis cubiti	+	-	-	+	-	-	+	+	+	+
Hypertrichosis of the back				+	+					
Hypertrichosis of limbs	+			+						
Hypertrichosis, generalized	+(limbs)	-	+	+	+	+, generalized	+	+	+	+, generalized
Thick hair	-	-		+	+	-	+	+	+	+
Thick eyebrow	+	-	+	+	-		+	+	-	+
**Development and neurology**										
Hypotonia	+	+, first year	+	-	+	-	+	-	+(mild)	+, first year
Intellectual disability	+	+	+	+	-	+	+	+	-	+
Speech delay/absence	+	+	+	+	+	+	+		+	+
Seizures	+	-	+, single event	-	-	-	+	-	-	-
Behavioral disorder	Wide-based gait	Wide-based gait	Aggressive behavior, wide-based gait, autistic features	Aggressive behavior, wide-based gait	Aggressive behavior(mild)	Aggressive behavior, wide-based gait	Wide-based gait, autistic features		Aggressive behavior(verbally), autistic features	
**Organic problems**										
Cerebral	Corpus callosum hypoplasia, cerebellar atrophy.		Cavum septum pellucidum, hypoplastic hippocampus, hypomyelination;							

Ophthalmologic examination	Other ocular anomalies(unknown)	+, severe myopia	-	-	-	-	-	-	+, myopia, astigmatism	+
Cardiac	-		-	+, ventricular septal defect, patent ductus arteriosus	-	+, patent ductus arteriosus	-	+, dense left ventricular trabeculae	+, heart murmur, resolved spontaneously	+, bicuspid aortic valve
Renal	+	-	+	-	+	-	-	-	+, right kidney dysplasia	+, horseshoe kidney
Feeding difficulties	-	+	+	+	’+（first year）	‘+（in infancy）	+	-	+	+
GH deficit	Corpus callosum hypoplasia, cerebellar atrophy.		Cavum septum pellucidum, hypoplastic hippocampus, hypomyelination;							
Other										

Wiedemann-Steiner syndrome patients with detailed clinical data and mutation in KMT2A

Study	Ramirez-Montano and Pachajoa 2019	Negri et al. 2019	Feldman et al. 2019		Chen et al. 2019	Chan et al. 2019					
Sample ID			Patient 1	Patient 2		Patient 1	Patient 2	Patient 3	Patient 4	Patient 5	Patient 6
Gender	F	M	F	M	M	M	F	M	M	M	M
Age (or age at last examination)	21m	6y	23y	2y	1y	10y	12y2m	13y	25y	5y1m	5y9m
Country/Race	Southwest Colombia		Western European	Caucasian	Chinese	Caucasian	Caucasian	Caucasian	Caucasian	Caucasian	Caucasian
Inheritance	De novo			De novo	De novo	De novo	De novo	De novo	De novo	De novo	De novo
cDNA	c.4177dupA	c.5363+1delG	c.6079+1G>C, p.IVS23+1G>C	c.173dupC	c.3929_3930delins A	c.10324delG	c.7087_7090del	c.6169del p.(Val2057Tyrfs*18)	c.7695_7696del	c.8543 T > C	c.8095 C > T
Protein				p.A59GfsX88	p.Pro1310Glnfs*46	p.Ala3442Profs*17	p.(Ser2363Leufs*12)	Maternally inherited PAX1 p.(G166A)d, e	p.Glu2566Lysfs*14	p.Leu2848Pro	p.Arg2699*
Current height	72 cm(-3.83SD)	112cm(10°)	150.6cm(4th)								
Current weight	8.7kg(-2.2SD)	22kg(50°)	76.1kg(86th)								
Current head circumference		52cm(50°)	56.7cm(75th)	16m,44.5cm(-2.34SD);24m,45.0cm(-2.58SD)							
**Growth**											
Prenatal growth retardation		+		+	-	+	-	-	-	-	-
Postnatal growth retardation		+	+		+	+	+	+	+	_	+
Developmental delay	+		+	+	+	+	+	+	+	+	+
**Craniofacial features**											
Microcephaly		+		+		+	-	+	-	+	-
High forehead						+, slight	-	-	+	+	+
Low hair line		+	+			+	+	+	-	+	+
Narrow palpebral fissures		+				+	+	+	+	+	+
Downslanting palpebral fissures	+	+	+			+	+	-	+	+	+
Hypertelorism	+	+				+	+		+	+	+
Strabismus		-				+	+		-	+	-
Eversion of lateral third of lower eyelids		-				+, mild	-	-	-	-	-
Long eyelashes	+	+	+			+	+	+	+, slightly long,thick	+	+
Ptosis		-	+			-	+	-	-	+	-

Broad and arching eyebrows			+			+	+	+	-	-	+
Synophrys	+	+	+								
Depressed nasal bridge		-									
Wide nasal bridge					+	-	+	+	+	-	+
Broad nasal tip		+	+			+	-	+	+	+	+
Bulbous nose											
Long philtrum	+	-	-								
High palate		+	+			+	+		+	+	+
Cupid's bow, exaggerated						+, slight	-	-	-	-	+
Low-set ears		+	+, small auricular left ear tag, left low-set and posteriorly rotated ear								
Downturned corners of the mouth											
Thin upper lip		+			+	+	+	+	+	+	+
Dental/oral anomalies		+, microstomia	+			+, dental crowding	+, early eruption of teeth	-	+, crowded	+, early eruption of teeth	+, early dentition
Other Craniofacial features	Round facies, dennis morgan folds, excessive thick facial hair	Narrow forehead, convex nasal bridge with low hanging columella, short philtrum, facial grimacing, micrognathia, and posteriorly angulated ears	Short philtrum, narrow jaw, telecanthus, marked posteriorly sloping forehead, micrognathia	Plagiocephaly without cranial suture ridging, slightly upturned nose, neck pterygia		Micrognathia, long palpebral fissures, prominent ear(slight (left>right)), auricular deformity, abnormal dermatoglyphics	Eyebrow lateral flare, long palpebral fissures, abnormal dermatoglyphics	Micrognathia, eyebrow lateral flare, long palpebral fissures, epicanthus, short columella, prominent ear, auricular deformity	Micrognathia, eyebrow lateral flare, prominent ear	Eyebrow lateral flare, long palpebral fissures, short columella, prominent ear, auricular deformity	Micrognathia, eyebrow lateral flare, long palpebral fissures, short columella
**Skeletal anomalies**											
Advance bone age		-				-	+	-			-
Delayed bone age		-	-								
Scoliosis		+					-	-	-	-	-

Small and puffy hands and feet			+, edema of the hands and feet			+	-	-	+, small hand	+, small hand, small foot	+
Brachydactyly		+	+			-	-	-	-	-	+
Clinodactyly		+	+			+	-	-	+	+	+
Rib anomalies							+, Klippel Feil Syndrome	+, Klippel Feil Syndrome	NA	-	+, Klippel Feil
Sacral dimple							-	+	-	+	-
Other skeletal anomaly	Broad feet, bilateral congenital hip dislocation	Fetal fingertip pads, broad thumbs, broad halluces, kyphosis, pectus excavatum	Left single transverse palmar crease			Mildly restricted flexion of the elbows, prominent digit pads, slim and muscular build, 2-3 toe syndactyly, broad first digits, long 2nd toe	Prominent digit pads, 2-3 toe syndactyly, broad first digits, long hallux	Joint laxity, prominent digit pads, slim but lanky, broad first digits	Contracture of the DIP joint, prominent digit pads, 2-3 toe syndactyly, broad first digits, tapering fingers, long hallux	Fusion of C2-C3 bodies, joint laxity, prominent digit pads, slim and muscular build, 2-3 toe syndactyly, broad first digits	Contracture of the DIP joint, occipitocervical junction stenosis, hip joint dislocation, prominent digit pads, slim and muscular build, 2-3 toe syndactyly, broad first digits, tapering fingers, long hallux
**Hairiness**											
Hypertrichosis cubiti			+		+	+	+	+	+	-	+
Hypertrichosis of the back	+		+			+	+		+	+	+
Hypertrichosis of limbs			+								
Hypertrichosis, generalized	+, generalized,back and around the mammillae		+			+	+	+	+	+	+
Thick hair					+	+	+	+	-	+	+
Thick eyebrow	+	+	+		+	+	+	+	+	-	+
**Development and neurology**											
Hypotonia	+, mild	+		-	+	+	+	+	+	+	+
Intellectual disability		+, mederate	+			+	+	+	+	+	+
Speech delay/absence	+	-			+	+	+	+		+	+
Seizures		+			-	-	+	+	-	-	-
Behavioral disorder	+, irritability without hyperactivity	-	+, aggression, anxiety, and autism spectrum disorder			+, autism spectrum disorder, anxiety, attention concerns,	+, anxiety, emotional regulation concerns,	+, autism spectrum disorder, anxiety, attention concerns,	+, autism spectrum disorder	+, autism spectrum disorder, attention concerns,	+, autism spectrum disorder, anxiety;Attention concerns, emotional

						emotional regulation concerns, externalizing behaviours	externalizing behaviours	externalizing behaviours		emotional regulation concerns, externalizing behaviours	regulation concerns, externalizing behaviours
**Organic problems**											
Cerebral	-	-	-	-	+	+, Corpus callosum short dimension in SP diameter and thinning of body; small/hypoplastic olfactory nerves	-	+, incomplete myelin maturation and hypoplastic olfactory nerves		+, pineal and pituitary cysts, dysplastic corpus callosum and fornices, delayed myelination, hypoplastic optic nerves and optic chiasm	+, pineal cyst
Ophthalmologic examination											
Cardiac		-	-			-	+, ventricular septal defect, resolved	-	-	-	+, ventricular septal defect,resolved
Renal	+, urinary infection and pyelonephritis	+	-					+		-	-
Feeding difficulties											
GH deficit	-										
Other	Moderate gastroesophageal reflux	Flammeus nevus/angioma, cryptorchidism	Sleep apnea/ sleep disturbances, late onset adrenal hyperplasia, hyperlipidemia, Irritable bowel syndrome	Craniovertebral junction anomalies	Succinylcarnitine and methylmal-onylcarnitine (C4DC) and 3-hydroxyisovalerylcarnitine (C5OH) mildly increased	Poor sleep, constipation, cryptorchidism	Poor sleep, constipation	Constipation	Disrupted sleep in childhood, unilateral constipation	Constipation, nasogastric or percutaneous endoscopic gastrostomy, cryptorchidism	Poor sleep, constipation

Wiedemann-Steiner syndrome patients with detailed clinical data and mutation in KMT2A

**Study**	**Enokizono et al. 2017**	**Bogaert et al. 2017**			**Aggarwal, Rodriguez-Buritica et al. 2017**	**Stellacci, *et al.* (2016)**	**Miyake, *et al.* (2016)**					**Yuan et al. 2015**
Sample ID		Family A II:2	Family A II:3	Family A I:2			patient 1	patient 2	patient 3	patient 5	patient 6	CdLS-3
Gender	M	M	M	F	M	M	M	F	M	F	M	F
Age (or age at last examination)	12y2m(6y)	11y	11y	46y	5y	14y	3y	9y2m	4y5m	3y	9y6m	1y9m
Country/Race	Japanese					Italian	Japanese	Japanese	Austrinian	Japanese	Japanese	Turkish
Inheritance	De novo	Inherited from affected mother	Inherited from affected mother	Unknown (affected mother)	De novo	De novo	Unknown	De novo	De novo	De novo	De novo	De novo
cDNA	c.3130_3133del	c.10835+1G>A	c.10835+1G>A	c.10835+1G>A	c.152_186del	c.3481T>G	c.7438C>T	c.6781C>T	c.3566G>A	c.1038del	c.2148del	c.2233C>T
Protein	p.Q1045P fs * 48	IVS28+1G>A	IVS28+1G>A	IVS28+1G>A	p. Pro51Argfs*84	p.Cys1161Gly	p.R2480*	p.Q2261*	p.C1189Y	p.V347Lfs*53	p.L717Cfs*39	p.R745*
Current height	106.7cm(-1.4SD)				90.7cm(<5th,-3.8SD)	-6SD						
Current weight	23.9KG(1.3SD)				13.6kg(<5th,-2.5SD)	-4SD						
Current head circumference					51cm(25-50th)	-6SD						
**Growth**												
Prenatal growth retardation	+				+	+	+	+	+	-	-	+
Postnatal growth retardation	-	+	+	+	+	+	+	+	+	+	+	
Developmental delay	+	-	+	-	+	+	+	+	+	+	+	
**Craniofacial features**												
Microcephaly		-	-	-		+	-	-	+	-	-	+
High forehead							+	+	-	+	-	
Low hair line							-	+	+	+	-	+
Narrow palpebral fissures	+	+	+	+	+	-	-	-	+	+	+	+
Downslanting palpebral fissures	+	+	+	+	+	+	+	-	+	+	+	+
Hypertelorism	+	+	+	+	+	+		-	-	+	+	+
Strabismus	+	-	-	-	+	+	+	-	-	-	+	+
Eversion of lateral third of lower eyelids							+	-	-	-	+	
Long eyelashes					+	+						+
Ptosis					+(left> right)		+	-	-	+	-	
Broad and arching eyebrows	+						+	-	-	+	+	+
Synophrys						+						+
Depressed nasal bridge						+						+
Wide nasal bridge		+	+	+		+	+	+	+	-	+	
Broad nasal tip	+	+	+	+		-	+	+	+	-	-	

Bulbous nose						+						
Long philtrum		-	-	-	+	-						+
High palate		+	+	+		-	+	+	-	-	-	+
Cupid's bow, exaggerated							+	-	-	+	+	
Low-set ears	+	+	+	+	+	-						
Downturned corners of the mouth					+							+
Thin upper lip		+	+	+	+	+	-	+	+	+	+	+
Dental/oral anomalies		-	-	-		+	+	-	+	+	+	+
Other Craniofacial features		Mild macrocephaly, micrognathia, telecanthus	Mild macrocephaly, micrognathia, telecanthus	Mild macrocephaly, micrognathia, telecanthus	Epicanthal folds, broad nose	Long palpebral fissures, epicanthus, short columella, prominent ear, depressed nasal tip, auricular deformity, micrognathia, abnormal dermatoglyphics	Depressed nasal tip	Eyebrow lateral flare, short columella, depressed nasal tip, abnormal dermatoglyphics	Micrognathia	Eyebrow lateral flare, long palpebral fissures, epicanthus, depressed nasal tip, micrognathia	Epicanthal folds, broad nose, micrognathia, dysmorphic ears	
**Skeletal anomalies**												
Advance bone age					-	-			-	-	-	
Delayed bone age					-							
Scoliosis							-	-	-	-	-	
Small and puffy hands and feet	+, puffy hands and feet	+	+	+		+, small hands and feet	-	-	+, puffy hands and feet	+, puffy hands and feet	+, small and puffy hands	+, puffy hands and feet
Brachydactyly												+
Clinodactyly	+	-	+	+		-	+	+	+	-	+	+
Rib anomalies							-	-	-	-	-	
Sacral dimple							-	+	-	+	-	
Other skeletal anomaly	Preaxial polydactyly(repaired), tapering fingers			Congenital hip dysplasia	Broad first digits, contracture of the DIP joint, prominent digit pads	Tapering fingers, 2-3 toe syndactyly, contracture of the DIP joint, joint laxity, prominent digit pads, slim and muscular build	Tapering fingers	Broad first digits, long hallux, prominent digit pads	Slim, muscular build	Preaxial polydactyly(repaired), tapering fingers		
**Hairiness**												
Hypertrichosis cubiti	+	-	-	-		+	-	+	+	+	+	
Hypertrichosis of the back		-	-	-	+	+	+	+	+	+	+	
Hypertrichosis of limbs												
Hypertrichosis, generalized		-	-	-		+	+	+	+	-	+	+
Thick hair		+	+	+		+		+	-	+	+	
Thick eyebrow	+	+	+	+	+	+	+	+	+	+	+	+
**Development and neurology**												
Hypotonia	+	-	-	-		+	+	-	+	-	-	
Intellectual disability		+	+	+	+	+	+	+	+	+	+	+
Speech delay/absence	+				+	+						
Seizures		-	-	-		+	-	-	-	-	-	
Behavioral disorder					+, sensitive to excessive noise	+, aggressive behavior)				+, hyperactivity	NA	

					and easily frustrated							
**Organic problems**												
Cerebral	+, hypogenesis of the corpus callosum			+, mild gliosis	-	-	-	-	+		+, hypogenesis of the corpus callosum	
Ophthalmologic examination	+, astigmatism											
Cardiac		+	+	-		+	+	-	-		-	+, patent ductus arteriosus
Renal	+	-	+	+		-	-	-	-		-	
Feeding difficulties		+	+	-		+	-	-	+	-	-	
GH deficit												
Other		Antibody deficiency, antibody deficiency, bronchiectasis	Antibody deficiency, antibody deficiency, bronchiectasis	Antibody deficiency, antibody deficiency	Subclinical hypothyroidism, snoring and obstructive sleep apnea(surgered)	Transient neonatal jaundice, cryptorchidism(treated), dolichocolon (long and large intestine with abnormal rotation), constipation, sleep disturbances, acute respiratory distress syndrome, congenital hypogammaglobulinemia, urinary trait infection	Poor sleep, constipation	Poor sleep, constipation, nasogastric or percutaneous endoscopic gastrostomy, recurrent otitis media	Constipation, deafness			Antibody deficiency, antibody deficiency, bronchiectasis

Wiedemann-Steiner syndrome patients with detailed clinical data and mutation in KMT2A

Study	**Dunkerton, *et al.* (2015)**		**Bramswig et al. 2015**	**Strom, *et al.* (2014)**		**Mendelsohn, *et al.* (2014)**	**Jones, *et al.* (2012)**				
Sample ID	Twin 1	Twin 2	K2431	patient 1	patient 2		WSS-1	WSS-2	WSS-3	WSS-5	WSS-6
Gender	F	F	M	F	M	F	M	F	F	M	F
Age (or age at last examination)	10y	11y	22y(12y)	9y	1y	4y3m	6y	8y	12y	8y	24y
Country/Race	Caucasian	Caucasian		Mexican	Caucasian (maternal) and Mexican (paternal)	Hispanic					
Inheritance	De novo	De novo		De novo	De novo	De novo	De novo	De novo	De novo	De novo	De novo
cDNA	c. 3247C>T	c. 3247C>T	c.3464G>A	c.4342T>C	c.4086+1G>A	chr11:118,339,487-118,355,089del	c.8806_8809del	c.8267del	c.6913del	c.7144C>T	c.4599dup
Protein	p.R1083*	p.R1083*	p.Cys1155Tyr	p.Cys1448Arg	IVS8+1G>A		p.Val2936*	p.Leu2756*	p.Ser2305Leufs*2	p.Arg2382*	p.Lys1534*
Current height			71cm(-4.46SD)	165 cm (>95th)	67 cm (<3rd)	94.8cm(0.85%)	0.4th–2nd	<0.4th	9th (GH)	9th	0.4th–2nd
Current weight				50 kg (95th)	6.24 kg (<3rd)		2nd–9th	<0.4th	25th	2nd	25th
Current head circumference			42cm(-4.6SD)		44.1 cm (5th)		9th	<0.4th	50th	<2nd	50th
**Growth**											
Prenatal growth retardation			+	-	-		-	+	+	+	-
Postnatal growth retardation	+	+	+	+	+	+	+	+	+	+	+
Developmental delay			+	+	+		+	+	+	+	+
**Craniofacial features**											
Microcephaly				-	-						
High forehead											
Low hair line			+		+	+					
Narrow palpebral fissures	+	+	-				+	+	+	+	+
Downslanting palpebral fissures				+	+		+	+	+	+	+
Hypertelorism	+	+		+	+	+					
Strabismus	-	-		+	-						
Eversion of lateral third of lower eyelids											
Long eyelashes			+	+	+		+	+	+	+	+
Ptosis			-			+					
Broad and arching eyebrows											
Synophrys	+	+	-	+							
Depressed nasal bridge			+								
Wide nasal bridge	+	+		+	+	+	+	+	+	+	+
Broad nasal tip	-	-		+	+		+	+	+	+	+
Bulbous nose				+							
Long philtrum	+	+	+								
High palate											

Cupid's bow, exaggerated							-	+	+	+	-
Low-set ears	+	+									
Downturned corners of the mouth											
Thin upper lip	+	+	+	+			+	-	-	-	+
Dental/oral anomalies				+		+					
Other Craniofacial features	Telecanthus, small ears, small ear canals	Telecanthus, small ears, small ear canals	Coarse face, broad nose, upturned nasal tip, large mouth, thick lower vermillion, macroglossia		Wide anterior fontanelle, right microphthalmia, micrognathia, telecanthus, two posterior hair whorls	Slightly low hanging columella, small ears	Eyebrow lateralflare	Eyebrow lateralflare			
**Skeletal anomalies**											
Advance bone age	-	-				+					
Delayed bone age											
Scoliosis			-				-	-	-	-	-
Small and puffy hands and feet	+, small hands/feet	+, small hands/feet		+, small feet and hands	+, doughy and redundant skin on her hands				+, small hands/feet	+, small hands/feet	
Brachydactyly			-								
Clinodactyly	+	+		+		+					
Rib anomalies						+, hypoplastic 12th ribs	+		+	+	-
Sacral dimple				+	+		+	+	+		+
Other skeletal anomaly			A/Hypoplasia of distal pha langes V, prominent interphalange al joints, prominent distal phalanges, hands V nail a/hypoplasia	Tapering fingers	Tapering fingers, broad 1st digit, 2-3 Toe Syndactyly, decreased muscle bulk, mild pectus excavatum, 3–4 partial left-hand syndactyly	Left hip with absence of the ossification center for the left femoral head and borderline dislocation of the right hip(treated)	Broadfirst digits, slim and muscular build	Tapering fingers, 2–3 toe syndactyly, long hallux, slim and muscular build	Broadfirst digits, tapering fingers, 2–3 toe syndactyly, long hallux, slim and muscular build	Tapering fingers	
**Hairiness**											
Hypertrichosis cubiti					-	-	+	+	+	+	+
Hypertrichosis of the back						+	+	+	+	+	+
Hypertrichosis of limbs						+	-	-	+	-	+
Hypertrichosis, generalized	+	+	+		-						
Thick hair			Sparse scalp hair								
Thick eyebrow	+	+	+	+	+	+	+	-	+	+	+
**Development and neurology**											
Hypotonia	+	+	+	+	+	+					
Intellectual disability			+	+		+					
Speech delay/absence											
Seizures			-								

Behavioral disorder			-				+, autism, aggressive behavior	+, autism	+, aggressive behavior	+, aggressive behavior	
**Organic problems**											
Cerebral			-			+, markedly decreased symmetric white matter volume loss and extensive thinning of the corpus callosum without evidence of hemorrhage					
Ophthalmologic examination			+, right retinal atrophy	+, correctable astigmatism							
Cardiac	+, patent ductus arteriosus	+, patent ductus arteriosus	+, patent ductus arteriosus, mitral valve prolapse				-	-	-	-	+, patent ductus arteriosus
Renal	+, small kidneys	+, small kidneys				+, grade IV vesicoureteral reflux and a left ureterocele					
Feeding difficulties			+	+		+	+	+	-	+	-
GH deficit											
Other			Frequent infections, cryptorchidism, fasciculation of tongue	Multiple urinary tract infections		Hypoxic-ischemic encephalopathy, recurrent urinary tract infections, poor bladder tone, gastroesophageal reflux, nissen fundoplication, gastrostomy tube placement, mildly decreased tone and lower limb reflexes	Constipation, nasogastric and percutaneous endoscopic gastrostomy feeding, poor sleep	Constipation, nasogastric and percutaneous endoscopic gastrostomy feeding, poor sleep		Constipation	

Wiedemann-Steiner syndrome patients with detailed clinical data and mutation in KMT2A

**Study**	**Carman et al. 2020**	**Arora et al. 2020**	**Stoyle, *et al.* (2018)**	**Mietton et al. 2018**	**Lebrun et al. 2018**				**Sun et al. 2017**		**Parenti, *et al.* 2017**
Sample ID					MWS1230	RTT4023	RTT4336	RTT4337	A.II-5	B.II-1	Patient 1
Gender	F	M	M	F	M	M	M	F	M	M	M
Age (or age at last examination)	1y11m	11m	8.6y(8.1y)	10y	11y	8y	4y	10y			15y
Country/Race	Turkey	India	White European						Chinese	Chinese	Turkey
Inheritance	De novo	De novo	De novo		De novo	De novo	De novo	De novo			De novo
cDNA	c.2312dupC	c.2632_2633del	c.7062delC	c.4189G > T	c.3460C>T	c.8558T>G	c.3581G>A	c.11322-1G>A p.(Lys3775Serfs*32)	c.8407C>T	c.2455C>T	c.8590C > T
Protein	p.Ser774Valfs*11	p.Arg878GLYfster*7	p.Ser2355LeufsTer18	p.G1397*	p.Arg1154Trp	p.Met2853Arg	p.Cys1194Tyr	IVS33-1G>A	p.Q2803*	p.Q819*	p.Gln2864*
Current height	80 cm (3rd–10th)	70cm(-1.8SD)	124.3cm(-0.8SD)		<-3DS	?	-3DS	0.5DS			155.6cm(-2.12SD)
Current weight	80 cm (3rd–10th)	6.8kg(-2.6SD)	52kg?		<-3DS	?	-2DS	2DS			49.7kg(-1.06SD)
Current head circumference	46.7 cm(10th–25th)	44cm(-1.39SD)			-3DS	-1DS	<-3DS	-1DS			53.5cm(-1.62SD)
**Growth**											
Prenatal growth retardation		+	+		+	-	-	-	+	+	
Postnatal growth retardation	+	+	+		+		+	-	+	+	+
Developmental delay	+	+		+	+	+	+	+	+	+	
**Craniofacial features**											
Microcephaly				+					+	+	+
High forehead									-	-	
Low hair line									+	+	+
Narrow palpebral fissures	+		+	+					+	+	
Downslanting palpebral fissures			+						+	+	
Hypertelorism	+	+	+	+	+	+	+	+	+	+	
Strabismus					+	-	+	+	-	+	
Eversion of lateral third of lower eyelids									+	+	
Long eyelashes		+	+		-	-	+	+	+	+	+
Ptosis			+						+	+	
Broad and arching eyebrows			+						-	-	+
Synophrys	+	+			+	-	+	+	-	-	+
Depressed nasal bridge		+							+	+	+
Wide nasal bridge	+				+	+	+	+	+	-	
Broad nasal tip									+	+	+
Bulbous nose					+	+	-	-	+	+	
Long philtrum	+	+							+	+	+
High palate	+								-	+	

Cupid's bow, exaggerated									+	+	
Low-set ears	+	+	+		+	+	+	+			
Downturned corners of the mouth									+	+	-
Thin upper lip	+				+	+	+	+	+	-	+
Dental/oral anomalies		+, small teeth			+	-	-	+, thick gums	-	-	
Other Craniofacial features	Round and flat face, short nose	Broad forehead, thick eyebrows with medial flaring;prominent antihelix, small nose, inverted V-shaped upper lip, micrognathia, prominent anti-helix	Ptosis and mild facial asymmetry	Plagiocephaly, small and dysplastic ears	Short palpebral fissures, flat face;Everted lower lip, myopia	Short palpebral fissures, flat face;Everted lower lip	Short palpebral fissures, flat face, narrow external auditory canals, everted lower lip, hypermetropia	Short palpebral fissures, flat face, narrow external auditory canals, everted lower lip, hypermetropia	Prominent digit pads	Prominent digit pads, epicanthus, eyebrow lateral flare, wide nose, anteverted nares, retrognathia, open mouth	Thick alae nasi, smooth philtrum
**Skeletal anomalies**											
Advance bone age											
Delayed bone age		+				-			+	+	-
Scoliosis				+					-	-	
Small and puffy hands and feet		+, small, soft fleshy hands			Small hands and feet	Small hands and feet	Larges and swollen hands and feet	+	+	+	+, small hands/ feet
Brachydactyly									-	+	-
Clinodactyly					+	-	+	+	-	-	+
Rib anomalies									-	+, pectus excavatum	
Sacral dimple	+								+	-	
Other skeletal anomaly	Tapered fingers				2-3 toe syndactyly	Tapering fingers	Tapering fingers, 2-3 toe syndactyly, atlas occipital and C2-C3 fusion, basilar impression	Hip joint dislocation, absent proximal transverse palmar crease	Hip joint dislocation, absent proximal transverse palmar crease	Syndactyly of toes	Tapered fingers
**Hairiness**											
Hypertrichosis cubiti		-							-	-	+
Hypertrichosis of the back									+	-	
Hypertrichosis of limbs	-										
Hypertrichosis, generalized	-	+(generalized)		+	+	+	+	+	-	-	+
Thick hair									+	+	
Thick eyebrow	+	+							-	-	+
**Development and neurology**											
Hypotonia	-	+			+	+	+	+	-	-	
Intellectual disability			+		+	+	+	+	+	+	+
Speech delay/absence	+				+	+	+	-			
Seizures		-			Generalized, tonic clonic	-	-	-	-	-	-

Behavioral disorder		Stranger anxiety, babbling	Autistic spectrum disorder	Hyperactivity, stereotypies, hyperventilation	Hyperactivity, stereotypies, sleep disorders	Stereotypies, sleep disorders	Stereotypies, sleep disorders		+		Stranger anxiety, babbling
**Organic problems**											
Cerebral	-	Aqueduct stenosis and dilated third and lateral ventricles	Hypoplastic pituitary gland	Chiari I	-	-	Chiari I	Ischemic encephalopathy	+	-	Aqueduct stenosis and dilated third and lateral ventricles
Ophthalmologic examination	-	-			Myopia		Hypermetropia;	Hypermetropia;			
Cardiac		Small atrial septal defect			+	-	-	-	Patent ductus arteriosus, patent foramen ovale	-	-
Renal		-	Vesico-ureteric reflux, mild hydronephrosis and hydroureter			-	-	-		-	Vesico-ureteric reflux, mild hydronephrosis and hydroureter
Feeding difficulties	+	+			+	+	+	-	+	+	-
GH deficit		+	+								
Other											

Wiedemann-Steiner syndrome patients with detailed clinical data and mutation in KMT2A

**Study**	**Jinxiu et al. 2020**	**Min Ko et al. 2017**		**Steel et al. 2015**	**Jiali Wang et al. 2021**	**Ling Ma et al. 2021**	**Yingxian Zhang et al. 2021**	**Lifang Dai et al. 2021**	**Huakun Shangguan et al. 2019**	**Huiqin Xue et al. 2021**	**Zhijie Gao et al. 2018**
Sample ID		Patient 1	Patient 2								
Gender	M	F	M	F	F	F	M	M	F	M	F
Age (or age at last examination)	10y	4.2y	8y(5m)	1y10m	5y4m	6y7m	11y	1y4m	5y	3y	4y
Country/Race	Chinese	Korean	Korean	Arabian	Chinese	Chinese	Chinese	Chinese	Chinese	Chinese	Chinese
Inheritance	De novo	De novo	De novo	De novo							
cDNA	c.1167–1170delAGAA	c.5932C>T	c.3504G>A	c.4906C>T	c.2318dupC	c.5005-2A>G	c.5803-3T>G	c.7071delC	c.10051delA	c.4906C>T	
Protein	p.Glu390Lysfs*10	p.Gln1978*	p.Gly1168Asp	p.R1636*	p.S774Vfs*12						p.C1189W
Current height		92.4 cm (-3.0SD)	63cm (-1.8SD)		102cm (<3rd)	109cm (-2.01SD)	110.2cm (<3rd)	66cm	100cm (-2.4SD)		
Current weight		13.0 kg (-2.6SD)	6.7kg (-1.6SD)		16kg (3-10th)	19.5kg (-0.8SD)	17kg	6.5kg	16kg (-1.09SD)		
Current head circumference		46.5 cm (-0.6SD)	41cm (-1.2SD)			47cm (-2.0SD)		42cm			
**Growth**											
Prenatal growth retardation		+				+					
Postnatal growth retardation	+	+	-	+	+	+	+	+	+		
Developmental delay	+			+							+
**Craniofacial features**											
Microcephaly						+					
High forehead											
Low hair line	+						+		+		
Narrow palpebral fissures	+	+	+		+			+			
Downslanting palpebral fissures					+						
Hypertelorism		+, mild	+	+	+	+		+	+	+	

Strabismus	+	+	-	+			+				
Eversion of lateral third of lower eyelids											
Long eyelashes					+		+	+			
Ptosis	+	+		+, compensatory retrocollis		+	+				
Broad and arching eyebrows							+				
Synophrys											
Depressed nasal bridge	+				+		+	+		+	
Wide nasal bridge	+	+	+			+			+		
Broad nasal tip		+	+								
Bulbous nose	+				+			+			
Long philtrum	+										
High palate	+										
Cupid's bow, exaggerated											
Low-set ears	+	+	+	+	+						
Downturned corners of the mouth	+				+						
Thin upper lip		+	+		+			+			
Dental/oral anomalies						+					
Other Craniofacial features	Telecanthus and telecanthus, frontalis suspension, amblyopia, external ear deformity	Short palpebral fissures, short nose	Short nose, external ear deformity	Epicanthic folds, anteverted nose	Epicanthic folds		Facial nevus				
**Skeletal anomalies**											
Advance bone age						+					
Delayed bone age					+		+		+		
Scoliosis					-	-	-		-		

Small and puffy hands and feet		+, small hands and feet	+	-							
Brachydactyly					+	+			+		
Clinodactyly		+	+								
Rib anomalies											
Sacral dimple								+			
Other skeletal anomaly		Developmental dysplasia of the hip	Simian line	Pectus excavatum, patent anterior fontanel, nonspecific myopathic pattern			Cubitus valgus				
**Hairiness**											
Hypertrichosis cubiti	+	+						+			
Hypertrichosis of the back	+					+	+	+	+		
Hypertrichosis of limbs	-		+		+	+	+		+		
Hypertrichosis, generalized					+	+	+		+	-	
Thick hair	+				+				+		
Thick eyebrow	+	+	+			+	+	+	+		
**Development and neurology**											
Hypotonia	+	-	+	+				-			+
Intellectual disability	+	+	+		+	+	+	+		+	
Speech delay/absence		+	+								
Seizures										+	-
Behavioral disorder								+, irritability, hyperactivity			
**Organic problems**											
Cerebral					-	-	-	+	-	+	-
Ophthalmologic examination				-				-			
Cardiac	Ventricular septal defects (treated)	+, patent ductus arteriosus	-	-		-	+	-	-	+	

Renal		-	-			-	-	+	-		
Feeding difficulties			+					+			
GH deficit					+	+					
Other		Left hip dislocation(treatde), fibromatosis colli	Disrupted sleep cycle, emotional lability, and aggressive behaviors	Gastrooesophageal reflux, obstructive sleep apnoea	recurrent respiratory tract infections						

Wiedemann-Steiner syndrome patients with detailed clinical data and mutation in KMT2A

**Study**	**Xuying Cao et al. 2019**	**Ka Chen et al. 2022**						**This study**	**Li et al. 2018**	**Baer, S. et al. 2018**	**Sheppard, S. E.. 2021**
Sample ID		patient 1	patient 2	patient 3	patient 4	patient 5	patient 6				
Gender	F	F	M	F	F	M	F	M	F8/M8	F11/M22	F52/M52
Age (or age at last examination)	6y6m	9.1y	11.2y	9.7y	1.0y	5.9y	5.2y	3y	1.5 to 25y	3 to 36y	
Country/Race	Chinese	Chinese	Chinese	Chinese	Chinese	Chinese	Chinese	Chinese	Chinese	French	
Inheritance	De novo	Unknown	Unknown	Inherited from father	Inherited from father	De novo	De novo	De novo			
cDNA	c.9762_9765del GATT	c.10900+2T>C	c.10837C>T	c.4332G>A	c.2508dupC	c.11695_11696d elinsT	c.9915dupA				
Protein	p. I3255Tfs*14	None	p.Gln3613*	p.E1444E	p.W838Lfs*9	p.T3899Sfs*73	p.P3306Tfs*22				
Current height	98.5cm(<3SD)	107cm	118cm	126.6cm	65.1cm	107.6cm	95.1cm	12kg			
Current weight	12.5kg	17kg	19.5kg	26.4kg	6.8kg	18.4kg	13.0kg	98.8kg			
Current head circumference	46cm										
**Growth**											
Prenatal growth retardation	+			+	+			-		13/31	
Postnatal growth retardation	+	+	+	+	+	+	+	+	13/16	17/32	
Developmental delay	+	+	+	+	+	+	+	+		24/30	
**Craniofacial features**											
Microcephaly		-	-	-	+	-	-	-	8/16	10/30	
High forehead								-	7/16		
Low hair line		+	+	+	+	+	+	+	13/16		
Narrow palpebral fissures								-			70/101
Downslanting palpebral fissures		+	+	+	+	+	+	+	12/16	18/31	51/103
Hypertelorism		+	+	+	+	+	+	-	13/16	21/32	63/94
Strabismus		-	+	+	-	-	-	-	3/14	7/32	36/96

Eversion of lateral third of lower eyelids								-			
Long eyelashes		+	+	+	+	+	+	+	15/16	24/32	72/101
Ptosis		+	+	+	+	+	+	-	10/16	5/32	40/93
Broad and arching eyebrows	+	+	+	+	+	+	+	+	6/16		
Synophrys								+	1/16		
Depressed nasal bridge		+	+	+	+	+	+	-	9/16		
Wide nasal bridge		+	+	+	+	+	+	-	10/16	22/31	64/101
Broad nasal tip								+			63/99
Bulbous nose								+			63/99
Long philtrum		+	+	+	+	+	+	-	9/16	20/32	5/95
High palate	+	+	+	+	+	+	+	-	12/16		
Cupid's bow, exaggerated								-			21/97
Low-set ears		+	+	+	+	+	+	-	6/16	15/30	
Downturned corners of the mouth								-	13/16		
Thin upper lip								-	8/16	24/32	47/96
Dental/oral anomalies	+	+	+	-	-	+	+	-	7/16	4/?	?(57.7%)
Other Craniofacial features	Micrognathia								1/16(macrocephaly), 2/16(epicanthus), 2/16(external ear deformity), 7/16(micrognathia), 1/16(cleft palate), 1/15(glossoptosis)	23/32(small palpebral fissures)	42/88 (lateral eyebrow flare), 7/103(up-slanted palpebral fissures), 4/94(hypotelorism), 7/89(bifid uvula), 28/99(posteriorly rotated ears)
**Skeletal anomalies**											
Advance bone age				-				-	2/10	7/15	7/29
Delayed bone age		+	+			+	Normal	-	7/10	5/15	4/29
Scoliosis								-	1/16	1/?	20/94
Small and puffy hands and feet								-	9/16	1/?	

Brachydactyly		+		+		+		-	8/16	9/29	
Clinodactyly								-	4/16	6/28	
Rib anomalies								+		4/17	21/62
Sacral dimple		+	+	+	+	-	+	-	4/16	8/25	46/91
Other skeletal anomaly		Increased palm lines	2-3 toe syndactyly, increased palm lines		2-3 toe syndactyly	Increased palm lines		flat-footed	1/16(deep palmar crease), 3/16(syndactyly), 1/13(carpal epiphyseal growth retardation), 2/16(absent palmar proximal transverse creases)	9/29(tapering fingers), 5/14(vertebral block), 1/?(club feet), 1/?(large hallux), 1/?(coxa vara pes planovalgus), 1/?(c1 posterior arch hypoplasia), 1/?(IInd toes clinodactyly), 1/?(II-III syndactyly), 1/?(hallux valgus), 1/?(pectus excavatum)	
**Hairiness**											
Hypertrichosis cubiti			+	+		+	+	+	7/16	19/31	57/100
Hypertrichosis of the back		+	+	+	+	+	+	+	12/16	21/31	68/101
Hypertrichosis of limbs		+	+	+	+	+	+	+	8/16	9/24	44/97
Hypertrichosis, generalized		+	+	+	+	+	+	+			
Thick hair		+	+	+	+	+	+	+	14/16		
Thick eyebrow								+	6/16	23/29	77/102
**Development and neurology**											
Hypotonia								-		18/31	63/87
Intellectual disability	+	+	+	+	+	+	+	+	14/15	33/33	96/99
Speech delay/absence	+	+	+	+	+	+	+	+	12/14	24/30	
Seizures								-		4/31	15/75
Behavioral disorder								-	4/16(aggressive behavior), 2/16(hyperactivity), 1/16(autism)	10/31	20/94(autism), 31/94(aggressive behavior), 39/88(hyperactivity)

**Organic problems**											
Cerebral		+	+		+, widening of the extracerebral space and reduction of periventricular white matter	-	-	-		10/29	30/52
Ophthalmologic examination								-	1/14(hyperopia)	9/32(hyperopia)	17/83(astigmatism)
Cardiac	-	-	-	-		+	+, sinus arrhythmia	-	3/16	8/22	29/81
Renal								-		7/23	36/77
Feeding difficulties								-	5/16	20/31	67/101
GH deficit						+		-	3/3	6/12	6/32
Other						Cryptorchidism		Cryptorchidism	1/16(dyslipidemia)	1/?(constipation), 1/?(rhinolalia cryptorchidis berger disease), 1/?(dyslipidemia)	42/91(Poor sleep);
